# Quality Evaluation of Ayurvedic Crude Drug *Daruharidra*, Its Allied Species, and Commercial Samples from Herbal Drug Markets of India

**DOI:** 10.1155/2013/472973

**Published:** 2013-01-22

**Authors:** Sharad Srivastava, A. K. S. Rawat

**Affiliations:** Pharmacognosy and Ethnopharmacology Division, National Botanical Research Institute, Lucknow 226002, India

## Abstract

*Berberis aristata* known as “*Daruharidra*” in Ayurveda is a versatile medicinal plant used singly or in combination with other medicinal plants for treating a variety of ailments like jaundice, enlargement of spleen, leprosy, rheumatism, fever, morning/evening sickness, snakebite, and so forth. A major bioactive marker of this genus is an alkaloid berberine, which is known for its activity against cholera, acute diarrhea, amoebiasis, and latent malaria and for the treatment of oriental sore caused by *Leishmania tropica*. Although the roots of *B. aristata* are considered as the official drug (Ayurvedic Pharmacopoeia of India), the study revealed that different species of *Berberis,* namely. *B. asiatica*, *B. chitria,* and *B. lycium* are also used under the name of *Daruharidra* in different parts of the country. Detailed physicochemical and phytochemical studies of subjects like total ash, acid insoluble ash, tannins, and total alkaloids were calculated from the shade dried powdered material according to the recommended procedures. Further, heavy metal studies and quantitative estimation of berberine through HPTLC have also been performed as per ICH guidelines. A detailed study of four *Berberis* species, namely *B. aristata*, *B. asiatica*, *B. chitria,* and *B. lycium,* which are implicated as *Daruharidra* and collected from wild and ten commercial samples procured from various important drug markets in India has been carried out, which may be useful to pharmaceutical industries for the authentication of the commercial samples and exploring the possibilities of using other species as a substitute of *B. aristata*.

## 1. Introduction 

*Berberis aristata* known as *“Daruharidra”* in Ayurveda is a versatile medicinal plant used singly or in combination with other medicinal plants for treating a variety of ailments like jaundice, enlargement of spleen, leprosy, rheumatism, fever, morning/evening sickness, and snakebite, and so forth [1–4]. In addition, the decoction of root or stem of *Berberis* known as “*Rasaut*” is specifically used in eye disease, skin disorders, and indolent ulcers. Its use in the management of infected wounds has also been described in Ayurvedic classical texts [5]. The major alkaloid of the plant is berberine, which is known for its activity against cholera [6], acute diarrhea [7], amoebiasis, and latent malaria and for the treatment of oriental sore caused by *Leishmania tropica *[4].

Although the roots of *B. aristata* are considered as the official drug [8], the study revealed that different species of *Berberis, namely, B. asiatica, B. chitria* and *B. lycium* are also used as *Daruharidra* in different parts of the country. In southern India, however, *Coscinium fenestratum* is used as “*Daruharidra.*” The study also shown that most of the market material sold as *Daruharidra *consists of mostly the stem parts than the roots of *Berberis* species.

As such there are different alkaloids available to differentiate different *Berberis* species. Several workers have also done molecular analysis of different *Berberis* species including the presented four species which reflects the use of molecular markers and sequence analysis for identification at inter- and intra-specific level [9–12].

Over exploitation of *B. aristata* created scarcity of the material that opened new vistas to identify a possible substitute for this species. During the market surveillance of different herbal drug markets of India, it was observed that almost all the markets either comprise *Berberis lycium *or *Berberis asiatica. *Although a detailed pharmacognostic study of *B. aristata, B. asiatica, *and *B. chitria* is reported by Srivastava et al. [13–15], market surveillance is not yet performed. Hence, the present study has been undertaken, which may be useful to pharmaceutical industries for the authentication of the commercial samples and to explore the possibilities of using other species as a substitute of *B. aristata*. 

## 2. Materials and Methods

The plant materials were collected from the Dhanaulti (Uttaranchal) region of India (LWG 221238-11) and the roots were preserved in 70% ethyl alcohol for histological studies. Procurement of commercial samples was done from various important drug markets of India, *namely,* Aligarh, Amritsar, Bangalore-I, Bangalore-II, Delhi, Hyderabad, Jammu, Lucknow, Trichur, Varanasi, and so forth.

Microtome sections were cut and stained with safranin and fast green and photographed with Nikon F70X camera [16]. Physicochemical and phytochemical studies like total ash, acid insoluble ash, tannins, and total alkaloids were calculated from the shade dried powdered material according to the recommended procedures [17–19]. Further, heavy metal studies and quantitative estimation of berberine through HPTLC have also been performed as per ICH guidelines.

## 3. Results and Discussion

**Figure 1:**
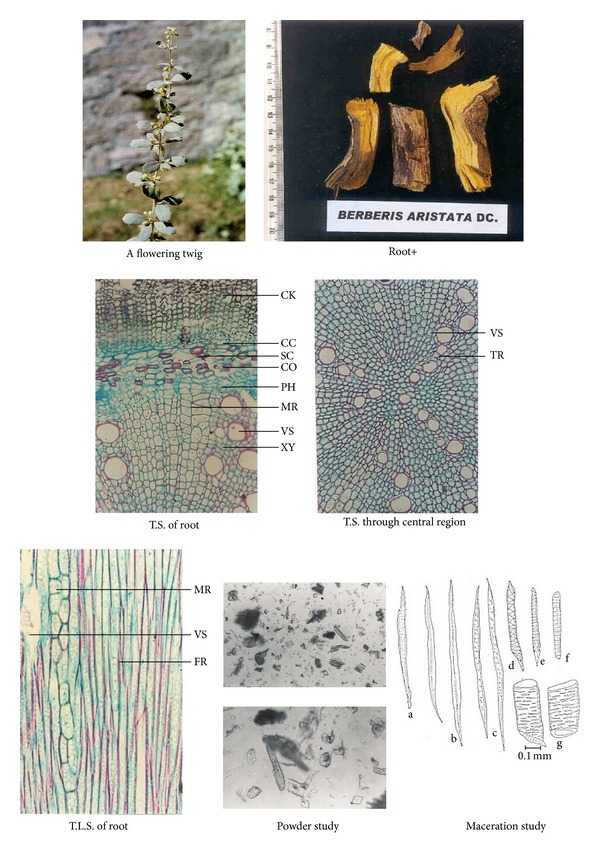
Anatomical characters of *Berberis aristata *root.

**Figure 2:**
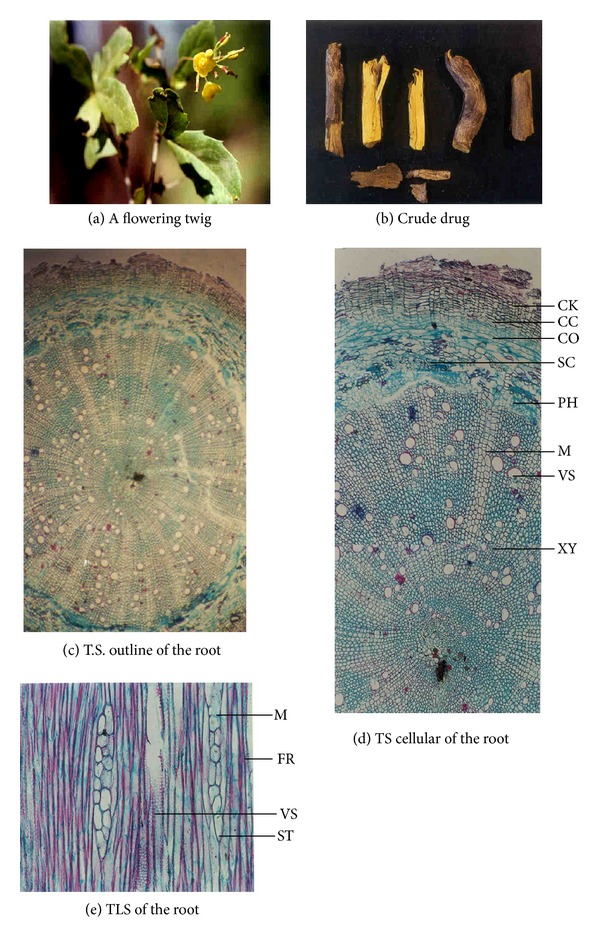
Anatomical characters of *Berberis asiatica *root.

**Figure 3:**
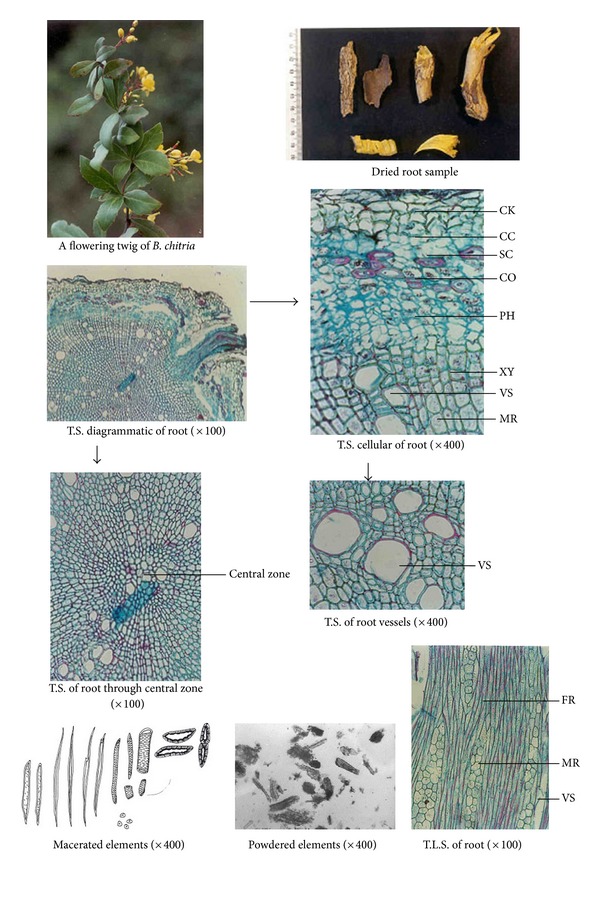
Anatomical characters of *Berberis chitria *root.

**Figure 4:**
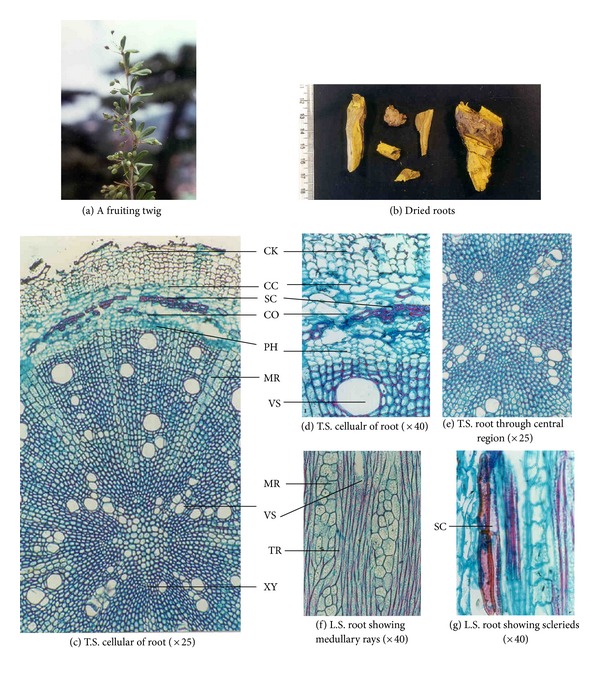
Anatomical characters of *Berberis lycium *root.

Morphological studies showed certain minor variations in all the four *Berberis* species (see Table 4). For example, in *B. aristata* and *B. chitria *the cut surface is bright yellow while that of *B. asiatica *and *B. lycium* is lemon yellow, and deep yellow, respectively. Similarly the colour of wood bark has also minor variation, namely, it is yellowish brown to yellowish gray in all the three species except in *B. lycium* the colour is grayish white. Likewise the numbers of pericyclic fibres are different in all the four species, for example, the maximum is found in *B. aristata* and minimum in *B. lycium* (Tables 1, 2, and 3; Figures 1, 2, 3, and 4).

**Figure 5:**
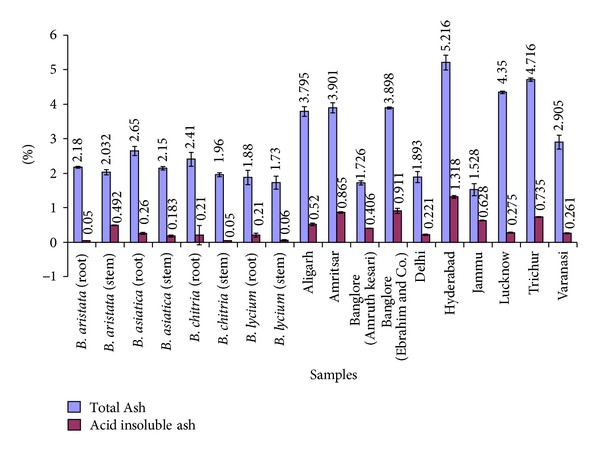
Comparative ash values of different *Berberis* species and market samples of *Daruharidra. *

**Figure 6:**
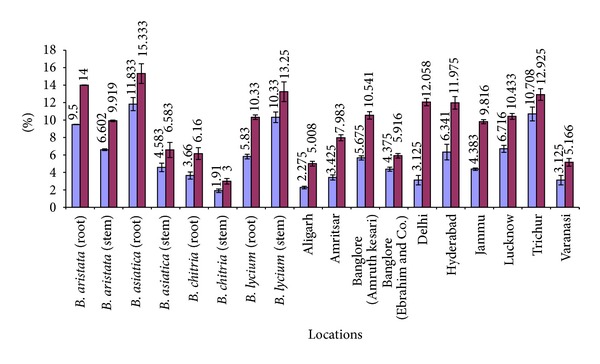
Comparative alcohol and water-soluble extractives values of different *Berberis* species and market samples of *Daruharidra. *

**Figure 7:**
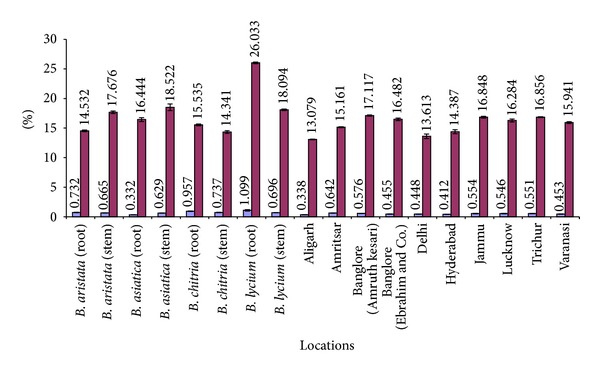
Comparative sugar and starch percentage of different *Berberis* species and market samples of *Daruharidra. *

**Figure 8:**
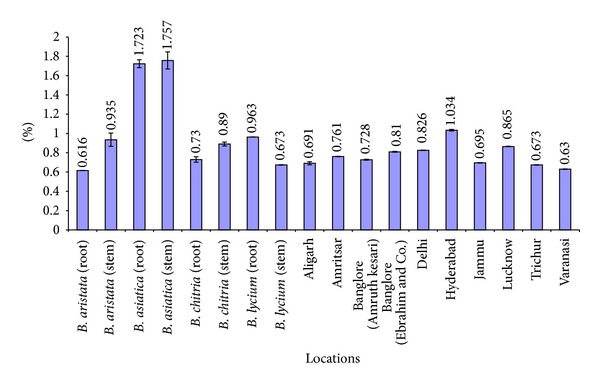
Comparative tannin percentage of different *Berberis* species and market samples of *Daruharidra. *

**Figure 9:**
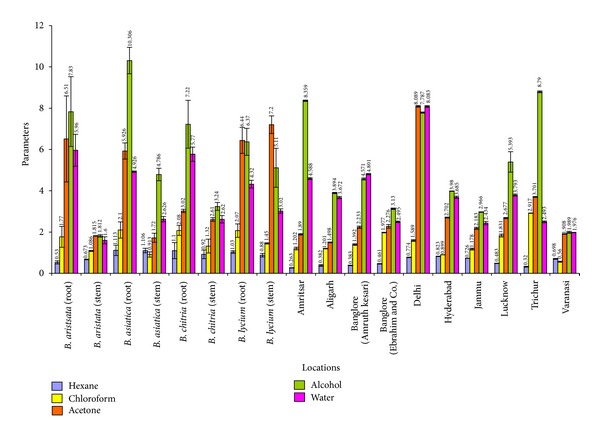
Comparative successive soxhlet extractive values of different *Berberis* species and market samples of *Daruharidra. *

**Figure 10:**
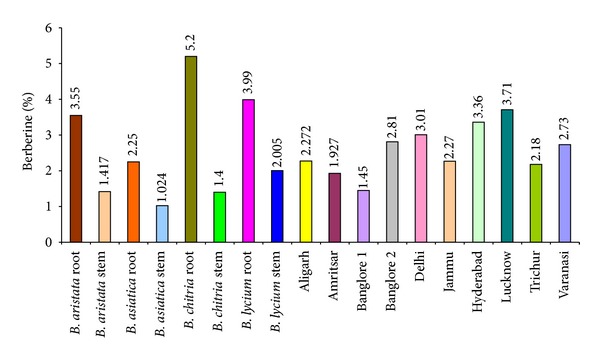
Quantitative estimation of berberine in different species of *Berberis *and market samples of *Daruharidra. *

A comparative account of all physicochemical values has been depicted in histograms (Figures 5–10). It is quite clear from these studies that no significant variation was observed in total ash of all the four species of *Berberis*. However, the percentage of acid insoluble ash of roots and stem showed significant variations; for example, the highest percentage of 0.26% acid insoluble ash was observed in *B. asiatica *root and the lowest one of 0.05% was noted in *B. aristata *root (Figure 5). It is interesting to note that the percentage of alcohol and water-soluble extractives were higher in root as compared to stem except in *B. chitria* (Figure 6). On the contrary the percentage of starch was higher in stem (14–19%) except in *B. lycium *(root) it was 26.03%. Percentage of tannin was more or less similar in both in root and stem of all the samples (0.7–1.7%).

Similarly, the percentage of successive Soxhlet extractive values revealed that alcohol, acetone, and water extractives were found to be significantly higher in *B. aristata* root, that is, 7.83%, 6.51% and 5.96% respectively. While the roots of *B. asiatica* possessed maximum percentage of alcoholic, acetone, and water-soluble matter, that is, 10.30%, 5.92%, and 4.92%, respectively. On the other hand the percentage of successive extractives in stem was maximum in *B. lycium* that is, 5.11% (acetone), 7.20% (alcohol), and 3.20% (water), respectively, and percentage of hexane soluble matter was higher in *B. aristata* (Figure 9).

The percentage of total crude alkaloid percentage was also estimated and it was found that it varied from species to species, that is, maximum in roots of *B. chitria* (3.65%) followed by the roots of *B. lycium* (2.8%), *B. aristata *(2.45%), and *B. asiatica* (2.4%), respectively. Besides, the active constituents berberine one of the major alkaloids was also calculated through HPTLC densitometric method (solvent system, n-propanol: water: formic acid, 90 : 80 : 0.4) and it was found more in roots as compared to stem, that is, 2.25–5.20% and 1.02–2.01%, respectively. Its concentration was also varied from species to species, that is, maximum in roots of *B. chitria* (5.20%) followed by *B. lycium* (3.99%), *B. aristata *(3.55%), and *B. asiatica* (2.25%). Details are depicted in Figure 10. 

**Figure 11:**
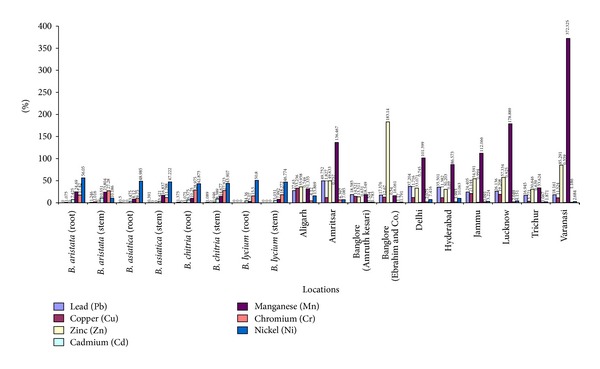
Comparative heavy metal studies of different *Berberis* species and market samples of *Daruharidra. *

**Figure 12:**
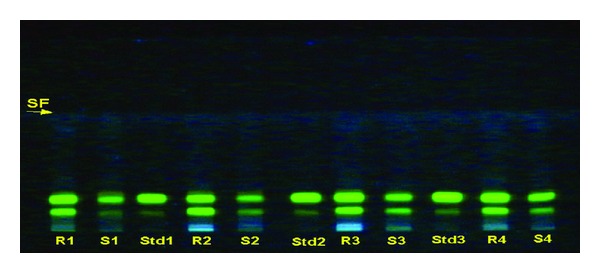
8 HPTLC profile of different *Berberis *species (under UV 366) (solvent system: n-propanol: water: formic acid, 90 :  80 : 0.4).

**Figure 13:**
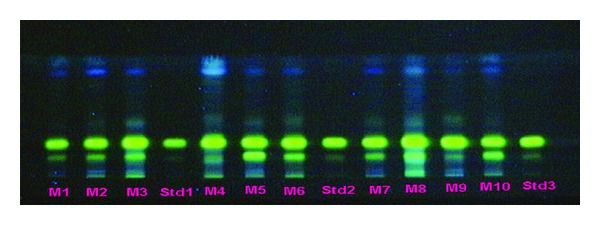
HPTLC profile of different market samples of *Daruharidra *(under UV 366); (Solvent system: n-propanol: water: formic acid, 90 : 80 : 0.4).

**Figure 14:**
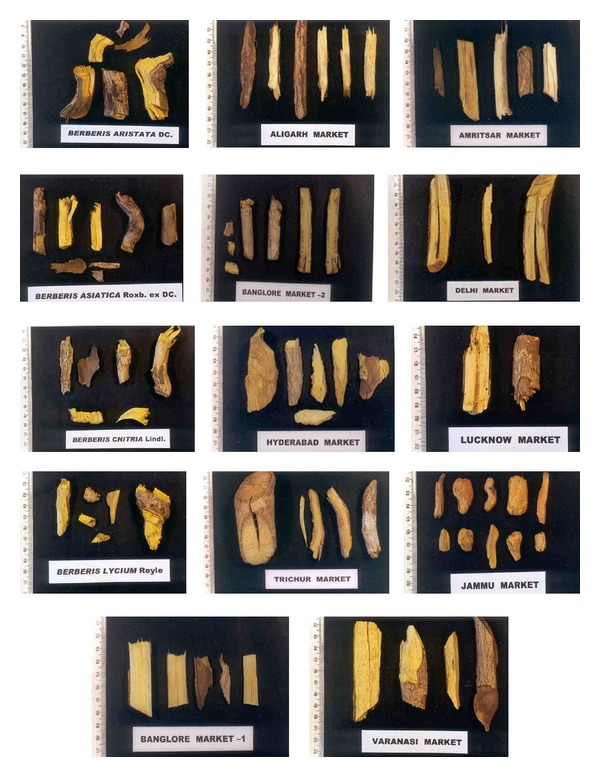
Crude samples of *Daruharidra *fromdifferent market of India.

**Figure 15:**
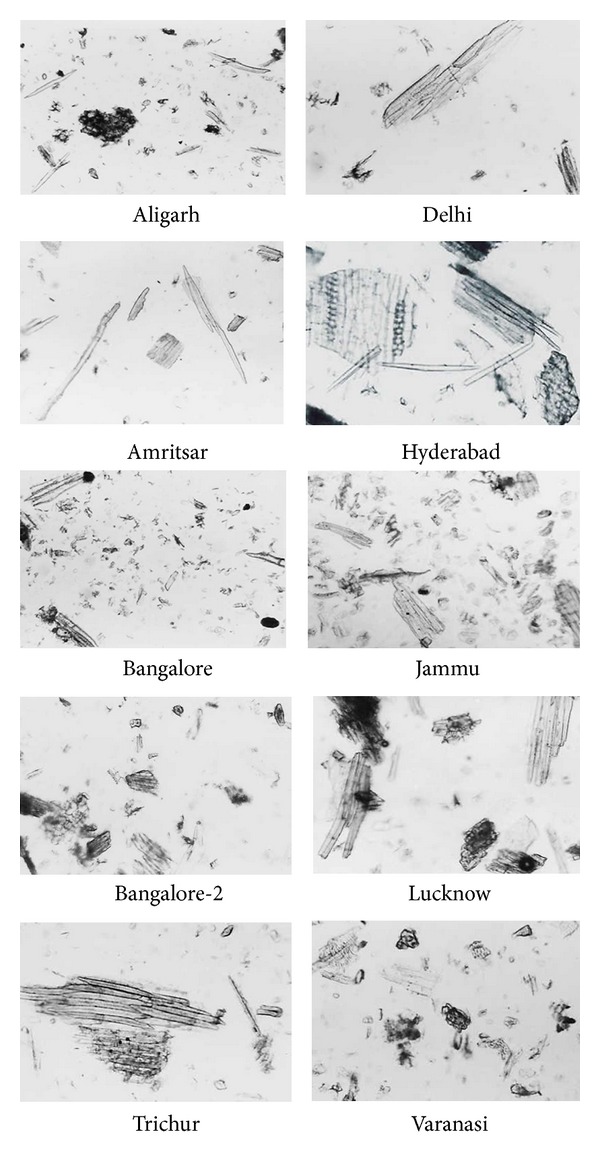
Powder study of different market samples of *Daruharidra. *

A comparative study of official drug *B. aristata* sample with that of commercial samples was made (Figures 5–15) and it was found that the Bangalore-I sample has all the similar morphological characters of roots of *B. asiatica, *namely, (i) outer surface grayish brown with 2 mm thick friable bark, which was separated out immediately leaving muddy yellow surface of the wood; (ii) transversely cut surface lemon yellow; (iii) sclerieds mostly in groups of 2–12 rarely solitary and comparatively more than other three species; (iv) pericycle fibres interrupted by stone cells; (v) length of the vessel elements much more than the other species, that is, up to 500 *μ*m (±181.0); (vi) physicochemical values are within the prescribed range (Table 6) of Ayurvedic Pharmacopoeia of India [9], hence, identified as roots of *B. asiatica*.

Similarly majority of anatomical characters of Aligarh and Varanasi samples matched with the stem and roots of *B. asiatica *in having (i) some pieces with fine longitudinal ridges and small warts on the outer surface of bark and dark brown outer surface of wood; (ii) transversely cut surface lemon yellow; (iii) sclerieds rarely solitary mostly in groups of 2–12 and comparatively more than other three species; (iv) pericycle fibres interrupted by stone cells; (v) length of the vessel elements much more than the other species up to 600 *μ*m (±181.0); (vi) trachieds up to 680 *μ* (±167.0) long; (vii) some other pieces have grayish brown outer surface with 2 mm thick friable bark which was separated out immediately leaving muddy yellow surface of the wood; presence of prominent pith as in stem of *B. asiatica*.

Furthermore, the commercial samples of Delhi and Lucknow showed close resemblance with the stem of *B. asiatica* by the presence of (i) fine longitudinal ridges and small warts on the outer surface of bark and yellowish creamy transverse cut surface; (ii) dark brown outer surface of wood as appeared after peeling off the bark; (iii) sclerieds rarely solitary mostly in groups of 2–12 and comparatively more than other three species; (iv) pericycle fibres interrupted by stone cells; (v) trachieds up to 680 *μ* (±167.0) and vessels up to 600 *μ* (±102.0) long (vi) pith.

Similarly, the market samples of Amritsar and Jammu were found to be the mixture of stem and root of two different *Berberis* species. Amritsar samples were found to be the stem of *B. aristata* and root of *B. asiatica *while Jammu sample comprised of root of *B. chitra *and stem of *B. asiatica*. 

The morphological characters in Amritsar sample are (i) outer surface creamish brown with knots, fine longitudinal ridges, and flakes; (ii) bark very thin and brittle; (iii) transverse cut surface bright yellow; (iv) outer surface of wood which appeared after peeling off the bark was yellowish brown; (v) sclerieds solitary or in a group of 2–10; (vi) pericyclic fiber mostly solitary, rarely in groups of 2–10; (vii) length of the fibres much more, that is, about 630 *μ*m as compared to other three species.

On the other hand some of the pieces of this sample showed close resemblance with that of the stem of *B. asiatica* in having (i) outer surface grayish brown with 2 mm thick friable bark which was separated out immediately leaving muddy yellow surface of the wood; (ii) transverse cut surface lemon yellow (iii) sclerieds rarely solitary mostly in groups of 2–12 and comparatively more than other three species (iv) pericycle fibres interrupted by stone cells; (v) length of the vessel elements much more than the other species, that is, up to 500 *μ*m (±102.0); (vi) presence of pith.

Similarly the morphological characters found in some pieces of Jammu samples showed close resemblance with those of stem of *B. asiatica*, as in Delhi and Lucknow markets. However, some other characters similar to roots of *B. chitria*. The different characters, this market samples showed are (i) outer surface light brown in colour with broad ridges and grooves and transverse marks; (ii) bark up to 5 mm thick but not easily detachable as in other three species; (iii) transverse cut surface bright yellow (iv) sclerieds mostly in groups of 2–4; (v) pericycle fibres present but are much lesser in number than *B. aristata *and *B. asiatica*; tracheidal fibres up to 960 *μ*m (±86.0) long.

The percentage of successive extractives (Soxhlet), sugar, starch, and berberine content also support the above finding (Figures 6–10).

On the contrary the Hyderabad commercial sample seems to be the mixture of three *Berberis *species. Some pieces showed close affinity with roots of *B. asiatica*; other pieces resembled roots of *B. aristata*. Besides some of the pieces have no resemblance with any of the four species studied. These may be the roots of *B. tinctoria* as it is a common species found in these areas.

The samples procured from Trichur and Bangalore II do not resemble any of the Berberis species studied. Presence of wedge shaped medullary rays and wheel shaped transverse section indicate that these pieces may belong to Menispermaceae. Hence an attempt was made to compare the characters of these samples with the root and stem of *Coscinium fenestratum*. After comparison it was found that samples procured from Trichur and Bangalore II were root and stem of *C. fenestratum* Gaertn, respectively.

From the heavy metal analysis of crude drug samples of different herbal market, it was observed that the concentration of majority of heavy metals, namely, Cd, Co, Mn, Zn, and Cu within the permissible limits of WHO in both the collected and commercial samples. However, the significant variations in Lead concentration were observed in both samples that is, in collected as well as in commercial ones. For example, in the stem of collected samples, the increase is quite low but in some of the commercial samples, namely, Amritsar, Delhi, and Hyderabad many fold increase in lead concentration was observed, this may be due to vehicular pollution or may be due to edaphic factors (Figure 11).

## 4. Conclusion

From the ongoing discussion it is quite clear that most of the commercial samples consist of mixture of roots or stems of *Berberis asiatica* while* B. aristata* is found only in the market samples of Amritsar and Hyderabad mixed with roots of *B. asiatica*.

On the basis of the above study it may be concluded that in India different *Berberis *species and their parts are being sold in the name of crude drug *Daruharidra* (Table 5). 

## Figures and Tables

**Table 1:** Comparative botanical analysis of the roots of four *Berberis *species.

Characters	*Berberis aristata *	*Berberis asiatica *	*Berberis chitria *	*Berberis lycium *
Macroscopic	Outer surface of the bark, creamish brown, and the inner surface attached to wood is yellowish brown. Bark 2 mm thick, knotty and brittle. Cut surface of the wood is bright yellow. Fracture hard, odourless and bitter in taste. Fine longitudinal ridges and flakes are present	Outer surface creamish brown but inner surface is muddy yellow. Bark 2 mm thick, friable separated out immediately from woody part. Cut surface of the wood lemon yellow. Fracture very hard, odour phenolic and very bitter in taste	Outer surface light brown, grooved with transverse marks, bark not easily detachable. Bark upto 5 mm thick, split longitudinally. Cut surface bright yellow. Fracture hard, odour faintly phenolic and very bitter in taste	Outer surface grayish brown with shinnings. Bark up to 3 mm thick, brittle, warty and easily detachable. Cut surface deep yellow Fracture hard, odour phenolic and bitter in taste
Cork cells	Brown, 10–20 layered	Brown, 12–15 layered	Dark brown, 8–10 layered	Dark brown, 8–11 layered
Cork Cambium	2 or 3 layered	1 or 2 layered	1 or 2 layered	2 or 3 layered
Cortical zone	30–35 layered, outer 4 to 6 layers compressed, devoid of stone cells	18–20 layered	12–20 layered	17–22 layered
Sclereids	Solitary or in group of 2 to 10	Rarely solitary but in group of 2 to 12, comparatively more than other three species	2 to 4 in groups	2 to 4 in groups
Pericyclic fibres	Mostly solitary but sometimes in groups of 2 to 10	Interrupted with stone cells	Frequently present comparatively lesser than *B. aristata* and *B. asiatica *	Frequently present comparatively lesser than other three species
Vessels	Solitary or in group of 2 or 3	Solitary or in group of 2 or 5	Solitary or in group of 2 or 3	Solitary or in group of 3 or 4
Medullary Rays	2 to 4 cells broad	2 to 3 cells broad	2 to 4 cells broad	2 to 5 cells broad

**Table 2:** Comparative Maceration study of the roots of four *Berberis* species.

Macerated elements (in *μ*m)	*Berberis aristata *		*Berberis asiatica *		*Berberis chitria *		*Berberis lycium *	
	Mean	SD	Mean	SD	Mean	SD	Mean	SD
Tracheids								
Length	491.024	±125.571	658.613	±253.672	348.308	±129.626	282.836	±4.598
Width	13.062	±4.600	13.749	±6.481	12.221	±2.645	12.603	±0.231
Vessels								
Length	259.720	±158.670	494.964	±181.477	160.405	±187.958	140.108	±14.862
Width	25.590	±18.198	20.623	±16.203	19.859	±11.533	24.629	±0.468
Fibres								
Length	645.481	±259.182	522.462	±246.290	476.632	±337.029	517.879	±2.217
Width	14.666	±3.986	11.457	±3.240	10.693	±2.645	10.884	±0.258
Tracheidal fibres								
Length	694.064	±324.992	714.949	±181.477	760.778	±194.440	368.931	±15.691
Width	12.986	±2.916	11.457	±3.240	11.457	±3.240	12.218	±0.250

**Table 3:** Comparative botanical analysis of the stems of four *Berberis *species.

Characters	*Berberis aristata *	*Berberis asiatica *	*Berberis chitria *	*Berberis lycium *
Macroscopic	Outer surface of bark, creamish brown, inner surface yellowish brown, knotty, thin, and brittle. Cut surface light yellow. Fracture hard and bitter in taste	Outer surface of bark grayish brown and friable, fine longitudinal ridges and small warts, inner surface dark brown. Fine longitudinal ridges and small warts below the bark surface leaving dark brown. Cut surface yellowish cream. Fracture very hard and very bitter in taste	Outer surface light brown, split longitudinally, warts comparatively large in size. Whole bark pealed off leaving coffee brown almost smooth inner surface. Cut surface light yellow. Fracture hard and bitter in taste	Outer surface grayish brown with shining. Bark easily detachable, thin, brittle, and twisted. Cut surface canary yellow. Fracture hard and bitter in taste
Cork cells	Brown, 15–25 layered	Brown, 08–10 layered	Dark brown, 8–15 layered	Dark brown, 7–19 layered
Cork Cambium	2 or 3 layered	1 or 2 layered	1 or 2 layered	2 or 3 layered
Cortical zone	20–25 layered, outer 4 to 6 layers compressed, devoid of stone cells	16–18 layered	20–24 layered	20–26 layered
Sclereids	Solitary or in group of 2 to 10	Sometimes solitary but in group of 2 to 4, comparatively more than other three species	Solitary	Scattered or sometimes in linear groups
Pericyclic fibres	Mostly solitary but sometimes in groups of 2 to 10	Interrupted with stone cells	Frequently present comparatively lesser than *B. aristata* and *B. asiatica *	Frequently present comparatively lesser than other three species.
Vessels	In group of 2 to 3 or solitary	Solitary or in group of 2 to 4	Mostly in group of 2 to 3 or solitary	Solitary or in group of 3 or 4
Medullary Rays	2 to 4 cells broad	2 to 5 cells broad	2 to 4 cells broad	1 to 3 cells broad
Pith	Present	Present	Present	Present

**Table 4:** Comparative Maceration study of the stems of four *Berberis* species.

Macerated elements	*Berberis aristata *		*Berberis asiatica *		*Berberis chitria *		*Berberis lycium *	
	Mean	Seed,	Mean	Seed,	Mean	SD	Mean	SD
Trachieds								
Length	439.03	±75.641	625.583	±169.764	360.398	±102.082	262.436	±3.145
Width	12.85	±4.600	12.417	±6.481	12.420	±2.645	11.603	±0.384
Vessels								
Length	459.72	±48.896	594.694	±102.728	468.908	±082.487	440.168	±14.862
Width	20.69	±10.186	18.623	±16.203	19.859	±11.503	20.629	±0.860
Fibres								
Length	627.38	±158.092	543.216	±180.780	424.426	±292.948	497.796	±2.217
Width	13.41	±3.986	11.246	±3.240	10.993	±2.645	18.443	±0.258
Tracheidal fibres								
Length	625.08	±224.887	706.843	±086.878	670.678	±094.087	334.632	±15.691
Width	11.49	±2.916	11.247	±3.240	11.457	±3.240	12.268	±0.250

**Table 5:** Market samples of different regions from India.

Serial number	Markets	Findings
1	Amritsar	Mixture of root of *B. asiatica * and stem of *B. aristata *
2	Aligarh	Mixture of root and stem of *B. asiatica *
3	Banglore I	Root of *B. asiatica *
4	Banglore II	Stem of *Coscinium fenestratum *
5	Delhi	Stem *B. asiatica *
6	Hyderabad	Mixture of root *B. asiatica* Root *B. aristata* Root of *B. tinctoria *
7	Jammu	Mixture of root of *B. chitria* and stem of *B. asiatica *
8	Lucknow	Stem of *B. asiatica *
9	Trichur	Root of *Coscinium fenestratum *
10	Varanasi	Mixture of root and stem of *B. asiatica *

**Table 6:** Identity, purity, and strength as prescribed by Ayurvedic Pharmacopoeia of India.

Parameters	Values
Foreign matter	Not *more* than 2 percent
Total ash	Not *more* than 14 percent
Acid-insoluble ash	Not *more* than 5 percent
Alcohol-soluble extractive	Not *less* than 6 percent
Water-soluble extractive	Not *less* than 8 percent
